# Metastatic Pattern, Local Relapse, and Survival of Patients with Myxoid Liposarcoma: A Retrospective Study of 45 Patients

**DOI:** 10.1155/2013/548628

**Published:** 2013-06-20

**Authors:** Hanna M. Fuglø, Katja Maretty-Nielsen, Dorrit Hovgaard, Johnny Ø. Keller, Akmal A. Safwat, Michael M. Petersen

**Affiliations:** ^1^Department of Orthopaedic Surgery, Rigshospitalet, University of Copenhagen, Blegdamsvej 9, 2100 Copenhagen Ø, Denmark; ^2^Department of Experimental Clinical Oncology, Aarhus University Hospital, Nørrebrogade 44, 8000 Aarhus, Denmark; ^3^Department of Orthopaedic Surgery, Aarhus University Hospital, Nørrebrogade 44, 8000 Aarhus, Denmark

## Abstract

*Purpose*. To assess the metastatic pattern of the histological subtype myxoid liposarcoma (MLS) with no or few round cells. *Methods*. Forty-five patients (F/M = 27/18, mean age 49 (range 17–85) years) were diagnosed with MLS at two Danish sarcoma centres in the period 1995–2004. A retrospective review of patients' files combined with an extraction of survival data from the Danish Centralised Civil Register was performed. *Results*. Seven patients had distant metastases during the observation period. Two patients had metastases at the time of diagnosis, while metastases occurred within 2.5 years in four patients, and in one patient 11.9 years after primary diagnosis. All metastases occurred at extrapulmonary sites. The first local relapse occurred within 3 years after surgery in six patients, in one patient after 4.0 years, and in one patient 7.7 years after surgery. The 5- and 10-year overall survival was 80% and 69%, respectively. Both the 5- and 10-year distant metastases-free survival was, respectively, 86%. The 5- and 10-year local relapse-free survival was, respectively, 83% and 80%. *Conclusions*. Patients with MLS had only extra-pulmonary metastases, and no lung metastases were found. Most local relapses and distant metastases occurred within the first 2-3 years after surgery.

## 1. Introduction

Sarcomas are very rare tumours and comprise approximately 1% of all newly diagnosed cancers [1]. The incidence is around 6 per 100,000 and has been rather constant during the last many years [2]. Liposarcomas are the second most frequent type of soft tissue sarcoma (STS), comprising 15–20% of all STS [3], and 30% of all liposarcomas are of the myxoid or myxoid round cell liposarcoma subtype [4, 5]. Originally, the myxoid and round cell liposarcomas (RCLS) were looked upon as two different histological subtypes but are now considered to represent a continuum of the same type, and the occurrence of areas with round cells is well known to be a poor prognostic factor [4].

STS has a tendency to, for most of the histological types, to metastasise haematogenously to the lungs whereas metastases to lymph nodes are uncommon [6]. Therefore, most guidelines recommend X-ray or computed tomography (CT scan) of the chest as the preferred method for evaluation of metastatic disease in the postoperative follow-up period [7–9]. Previously published studies have shown that the metastatic pattern in liposarcoma and especially myxoid liposarcoma (MLS) also includes extrapulmonary sites [10–18].

The purpose of this study was to evaluate the metastatic pattern, local relapse, and survival of patients with the classical subtype of MLS with none or few round cell areas.

## 2. Patients and Methods

Patients treated for a sarcoma with the histological diagnosis of MLS (only the classical type without or below 5% of round cells areas) in two Danish sarcoma-centers (the Department of Orthopaedic Surgery, Rigshospitalet, Copenhagen University Hospital, and the Department of Orthopaedic Surgery, Aarhus University Hospital) during the period from January 1, 1995 to December 31, 2004, were included in the study. In Copenhagen, the patients (*n* = 30) were identified using the local pathology database, and in Aarhus the patients were identified using the local clinical sarcoma database (*n* = 20). We excluded five of the patients from the Copenhagen material because the diagnosis of MLS was already present before January 1, 1995 (*n* = 3), the patient was never examined or treated at the Department of Orthopaedic Surgery (*n* = 1), and the final diagnosis was not an MLS (*n* = 1). Thus, a total of 45 patients (female/male = 27/18, mean age 49 years (range 17–85 years)) were left for the study with 18 patients diagnosed and initially treated in the period 1995–1999 and 27 patients in the period 2000–2004.

The anatomical location of the tumours was the thigh (*n* = 26), below knee or foot (*n* = 5), gluteal region or hip (*n* = 4), shoulder or upper extremity (*n* = 4), knee (*n* = 3), chest wall/neck (*n* = 2), and abdominal wall (*n* = 1). Thirty-eight patients had deep seated and 7 had superficial tumours. Information regarding tumour size (largest diameter) was available in 43 patients (measured by the pathologist (*n* = 36)), from preoperative magnetic resonance imaging (MRI) (*n* = 6) or a preoperative ultrasound examination (*n* = 1)) and the mean size was 11.8 cm (range 2.0 cm–34.0 cm). With the exception of one patient, all had surgical removal of the tumour as the initial treatment. The surgical margins obtained, evaluated according to Enneking's classification [19], was wide (*n* = 25), marginal (*n* = 13), intralesional (*n* = 4), or radical (*n* = 2). Nineteen patients were treated solely with surgery, while the remaining 25 patients were treated with surgery combined with external radiation therapy (*n* = 17), brachytherapy followed by external radiation therapy (*n* = 7), or chemotherapy (*n* = 1). In one patient, information regarding additional treatment was not available. All patients were followed routinely by clinical examination and X-ray or CT scan of the chest. If local recurrence was suspected or if the clinical examination for local recurrence was difficult due to, for example, complications from radiation therapy, an MRI was performed. Distant metastases were detected if visible on CT or X-ray of the chest or from relevant additional examinations performed because of symptomatic metastases. If a local recurrence or distant metastases were detected a new follow-up period was started after the treatment was completed.

### 2.1. Data and Statistics

We performed a retrospective review of the patients' files and collected data of demographics, anatomical localization of tumour including depth, histological description of tumour (histology, classification, surgical margin, and tumour size), imaging results from PET/CT, MRI, X-ray of chest and CT of chest, ultrasound scan, and bone scintigraphy performed at the initial workup and afterwards during clinical followup, which lasted a mean of 5.8 years (range: 0.1–15.8) for all patients (*n* = 45). Mean clinical followup for patients that had died (*n* = 15) or were still alive (*n* = 30) was, respectively, 3.4 years (range, 0.1–11.6) and 6.7 years (range, 3.0–15.8).

For evaluation of the metastatic pattern, local relapse, and survival, the routine case report was used, and the first distant metastasis and first local relapse was verified by the collected data of histology, diagnostic imaging at the time of diagnosis and during the followup. Data for patients' survival was obtained from the Danish Centralised Civil Register on June 1, 2011, and gave a mean time of followup for survival data of 4.7 years (range: 0.1–11.8) or 10.3 years (range: 6.6–15.8) for the patients that died during the followup (*n* = 15) or were still alive (*n* = 30), respectively, and the mean time of follow-up for all patients was 8.5 years (range: 0.1–15.8).

SPSS version 19.0 for Windows and Microsoft Excel version 2007 were used. Descriptive data are presented as mean and total range. The Kaplan-Meier survival analysis was used to determine the probability of 5- and 10-year overall survival, distant metastases-free survival, and local relapse-free survival after the primary diagnosis. In addition, the mean survival with 95% confidence interval (95%-CI) was calculated, and for comparison of overall survival between subgroups, logrank test was performed. For the calculation of overall survival, all deaths (regardless of the course of death) were considered an event in the survival analysis and the patients were censored at the end of followup (June 1, 2011). The event in calculation of distant metastases-free survival was occurrence of a distant metastasis, and the patients were censored if they had died or at the end of the followup (June 1, 2011). The event in calculation of local relapse-free survival was local relapse and the patients were censored if they died or at the end of the followup (June 1, 2011).

For evaluation of the influence of various clinical parameters on survival, the following subgroups were created: ± MLS distant metastases, ± local recurrence, 48 years ≥ age > 48 years, tumour depth (deep versus subcutaneous), tumour size (8 cm ≥ largest diameter > 8 cm), and surgical margin (radical or wide versus marginal or intralesional).

## 3. Results

### 3.1. Distant Metastases and Survival

**Figure 1:**
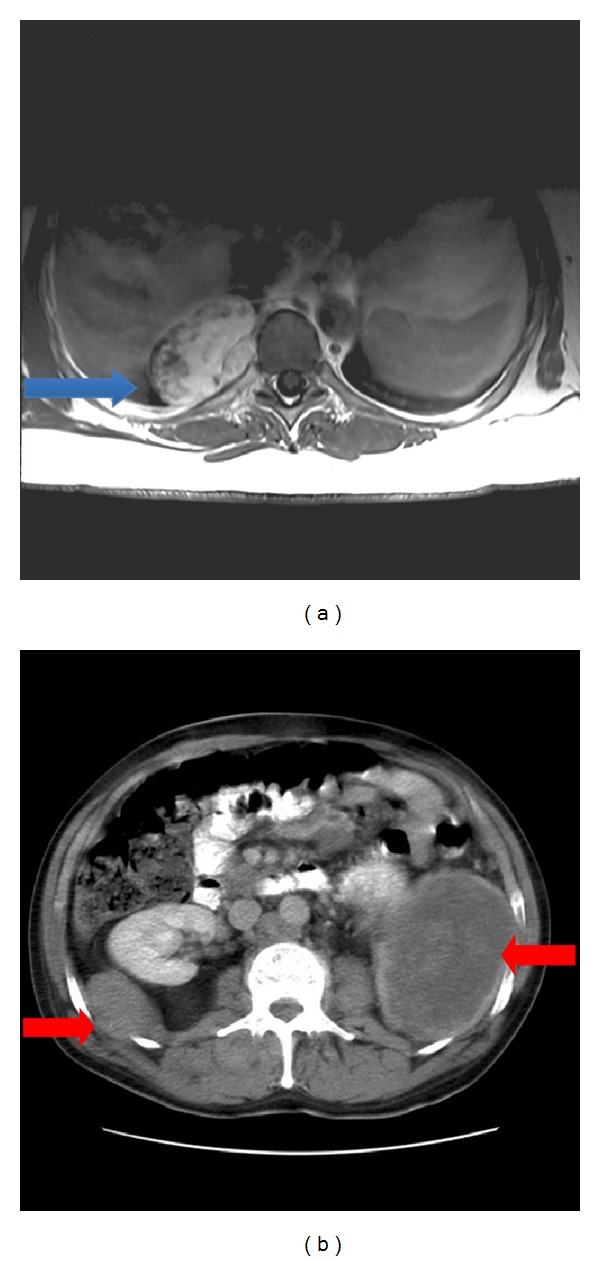
Magnetic resonance imaging showing a paravertebral metastasis of the thoracic cavity (blue arrow) just above the diaphragma diagnosed 12 years after operation for a primary tumour of the foot (and 6 months after a local relapse) (a). Abdominal computed tomography of a patient with primary tumour of the chest wall showing bilateral retroperitoneal metastases (red arrows) diagnosed at the same time as the primary tumour (b).

During the followup we found that 7 of 45 (16%) patients had or developed distant metastases on average 2.5 years (range, 0.0–11.9 years) after the primary diagnosis, and the mean followup of patients without metastases was 9.4 years (range, 0.1–15.2 years). All metastases were extrapulmonary and no patients had regular pulmonary metastases (Figure 1). In two of the 7 patients, the metastases were present at the time of initial diagnosis. The precise anatomical localisation of the metastases in the 7 patients is shown in Table 1. Moreover, distant MLS metastases were suspected in three additional patients: two patients developed metastases of, respectively, the lungs and of the spine and the liver from other cancers (malignant melanoma and breast cancer) during the followup, and in one patient a weak suspicion of bone metastases from bone scintigraphy and CT scan of pelvis could not be verified. The primary tumour in patients with metastases was in all cases deep seated and located at the thigh (*n* = 4), knee (*n* = 1), foot (*n* = 1), and chest wall (*n* = 1).

**Figure 2:**
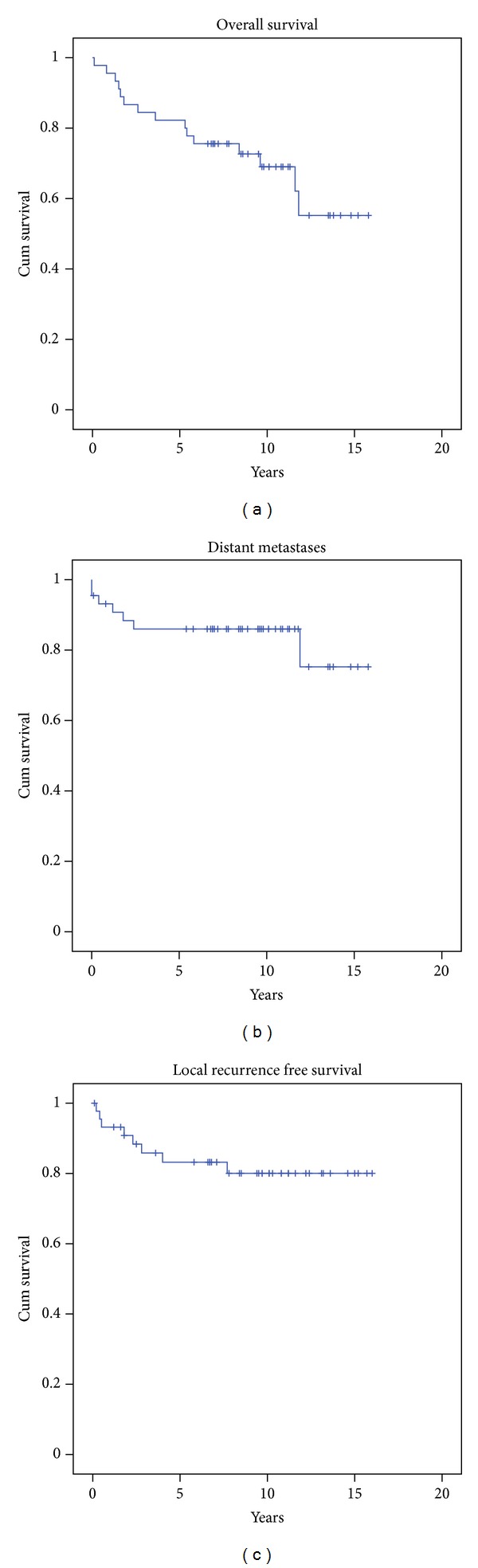
Kaplan-Meier survival curves for overall survival (a), distant metastases-free survival (b), and local recurrence-free survival (c).

Kaplan-Meier survival analysis showed that both the 5- and 10-year distant metastases-free survival could be estimated to 86% (Figure 2(b)) and the mean metastases-free survival was 13.3 years (95%-CI, 11.6–15.0 years). Fifteen (33%) patients died during the followup and a Kaplan-Meier survival analysis estimated that the 5- and 10-year overall survival was 80% and 69%, respectively (Figure 2(a)); the mean survival was 11.6 years (95%-CI, 9.9–13.3 years).

**Figure 3:**
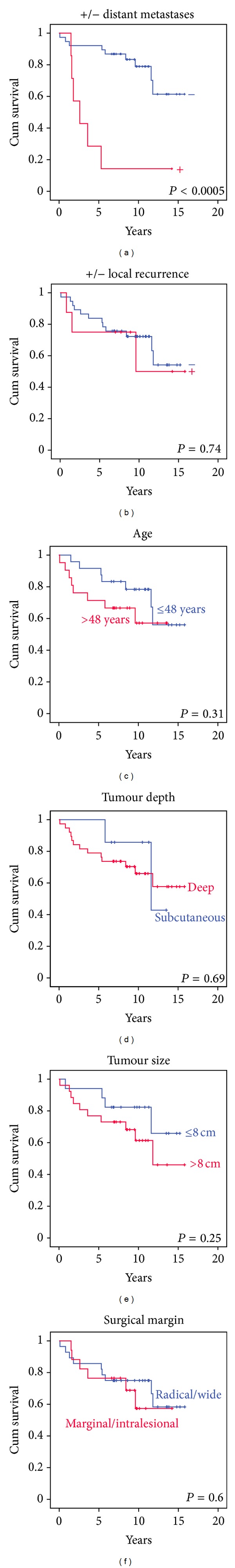
Kaplan-Meier survival curves with the result of logrank test for evaluation of the influence of various clinical parameters on survival.

When performing subgroup analysis evaluating the influence of various clinical parameters (distant metastases, local recurrence, age, tumour depth, tumour size, and surgical margin) on survival, the only significant parameter was distant MLS metastases (Figure 3). Mean survival in patients with (*n* = 7) and without (*n* = 38) distant MLS metastases was 4.4 (95% CI, 1.3–7.5 years) and 12.8 years (95% CI, 11.2–14.5 years), respectively.

### 3.2. Local Recurrence

We found that 8 of 45 (18%) patients developed a local recurrence on average 2.5 years (range, 0.3–7.7 years) postoperatively. The followup of the 37 patients without local recurrence was on average 8.8 years (range, 0.1–15.2 years) after the primary diagnosis. The anatomical locations where local recurrences occurred was on the shoulder (*n* = 1), forearm (*n* = 1), abdomen/lumbar region (*n* = 1), buttock (*n* = 1), thigh (*n* = 2), knee (*n* = 1), and foot (*n* = 1). Kaplan-Meier's survival analysis estimated the 5- and 10-year local relapse-free survival to 83% and 80%, respectively (Figure 2(c)), and the mean survival was 13.3 years (95% CI, 11.7–15.0 years).

## 4. Discussion 

We analysed 45 patients with MLS and found distant metastases in 7 (16%) patients. Compared to what is seen in STS in general, this is an unusual metastatic pattern, since all metastases occurred at extrapulmonary sites. However, a very similar anatomic metastatic distribution pattern as that seen in the present study has also been observed in other studies evaluating the results after treatment of patients with MLS [10–18].

Some of the previous studies also focused on the occurrence of round cells in the tumours, and in patients with tumours containing more than 5% of round cell areas, a significantly higher tendency towards distant metastases was found [10, 16, 17]. However, the distribution of the anatomic location of the distant metastases in these tumours was also mainly extrapulmonary [10, 16, 17], confirming the theory that the pure MLS and RCLS is now considered to represent a continuum of the same type, with the occurrence of areas with round cells considered to be a poor prognostic factor [4]. 

In the previously published studies [10–18], when focusing on tumours with pure myxoid histology or with no more than 5% round cell areas, the frequency of distant metastases varied from 5% to 32%. In our study, the percentage of patients suffering from distant metastases of 16% was close to the average value of previously published studies. The great variation in the percentage of patients suffering from distant metastases between studies could be explained by a significantly different length of followup and small sample sizes. 

If we focus on when distant metastases were diagnosed in the present study, we found that two patients had metastases at the time of diagnosis, while metastases occurred within 2.5 years after primary diagnosis in four patients, and after 11.9 years in one patient. All previously published studies [10, 12–15, 20] found as in the present study, that the majority of the first metastases occurred within the first 5 years after the diagnosis, but late occurrence of the first metastasis beyond 5 years was not unusual. 

In the present study, 15 (33%) patients died during followup, and the Kaplan-Meier survival analysis estimated a 5- and 10-year overall survival of 80% and 69%, respectively. Our results are consistent with four previously published studies reporting survival data as overall survival [11, 13–15, 18]. Our subgroup analysis evaluating the influence of various clinical parameters on survival showed that only the occurrence of MLS distant metastases significantly influenced survival. This was not a surprising finding, and it was confirmed by Spillane et al. [13]. However, when subgroups are as small as in the present study (and in most other studies evaluating MLS) the results of this kind of analysis should be interpreted with caution.

Trovik [21] reported the local recurrence rate in a large material of various types of STS located in the extremities or trunk wall, originating from the Scandinavian Sarcoma Group Register (*n* = 1613). In a subgroup of these patients (*n* = 1331) treated at a sarcoma centre, the local recurrence rate was 17%. We found that 8 of 45 (18%) patients developed a local recurrence after on average 2.5 years. Our local recurrence rate thus was on the same level as seen in STS in general [21], and in previously published papers evaluating the local recurrence rate after surgical treatment of MLS [10, 11, 13, 15, 18]. If we focus on when local recurrence after surgically removed MLS occurred, six patients had the first noted local recurrence within three years (range, 0.3–2.8 years), while one patient had the first local recurrence as late as 7.7 years postoperatively. In some of the previously published studies [10, 15], late local recurrence far beyond 5 years postoperatively was seen. 

## 5. Conclusion

We analysed 45 patients with MLS and found that 7 (16%) patients developed metastases. In all patients, the first metastatic lesion was extrapulmonary and in one case the first metastatic lesion occurred beyond 10 years postoperatively. Fifteen (33%) patients died during followup and the Kaplan-Meier survival analysis estimated a 5- and 10-years overall survival of 80% and 69%, respectively. We found that 8 (18%) patients developed a local recurrence after on average 2.5 years postoperatively, but a first local recurrence as late as 7.7 years postoperatively was seen in a single patient.

Based upon the findings of this study and our review of the literature, we recommend that in patients treated for MLS of the classical type, with no or few round cells, diagnostic imaging including both the lungs and abdomen/retroperitoneum (e.g., CT of thorax and abdomen) should be performed as a part of the postoperative control. It should be considered to extend the followup beyond five years postoperatively.

## Figures and Tables

**Table 1:** Data of the 7 patients with distant metastases.

Sex (age at diagnosis)	Anatomical location of primary tumour	Largest diameter of primary tumour	Surgical margin	Location of first distant metastases (time after diagnosis)	Alive/dead (time after diagnosis)
Female (84 years)	Thigh	16 cm	Radical	Bone metastases of the cervical, thoracal and lumbar spine (1.3 years)	Dead (1.8 years)
Male (45 years)	Thigh	34 cm	Marginal	Intraabdominal metastases (present at diagnosis)	Dead (2.5 years)
Male (58 years)	Chest wall	Unknown	Intralesional	Intraabdominal and multiple skin metastases (present at diagnosis)	Dead (1.6 years)
Female (22 years)	Thigh	15 cm	Intralesional	Bone (lumbar spine) and widespread intra- and paraspinal metastases (2.3 years)	Dead (3.7 years)
Male (43 years)	Foot	Unknown	Marginal	Paraspinal soft tissue metastasis of the thoracal spine (11.9 years)	Alive (13.2 years)
Female (29 years)	Knee	14 cm	Intralesional	Mediastinum and paraspinal soft tissue metastases of the lumbar spine (0.4 years)	Dead (1.4 years)
Male (39 years)	Thigh	14 cm	Radical	Solitary pleural metastasis (1.8 years)	Dead (5.3 years)
